# Advancements in single-cell RNA sequencing and spatial transcriptomics: transforming biomedical research

**DOI:** 10.3389/abp.2025.13922

**Published:** 2025-02-05

**Authors:** Getnet Molla Desta, Alemayehu Godana Birhanu

**Affiliations:** ^1^ College of Veterinary Medicine, Jigjiga University, Jigjiga, Ethiopia; ^2^ Institute of Biotechnology, Addis Ababa University, Addis Ababa, Ethiopia

**Keywords:** single-cell RNA-sequencing, transcriptome, high-throughput, spatial transcriptomics, technology development

## Abstract

In recent years, significant advancements in biochemistry, materials science, engineering, and computer-aided testing have driven the development of high-throughput tools for profiling genetic information. Single-cell RNA sequencing (scRNA-seq) technologies have established themselves as key tools for dissecting genetic sequences at the level of single cells. These technologies reveal cellular diversity and allow for the exploration of cell states and transformations with exceptional resolution. Unlike bulk sequencing, which provides population-averaged data, scRNA-seq can detect cell subtypes or gene expression variations that would otherwise be overlooked. However, a key limitation of scRNA-seq is its inability to preserve spatial information about the RNA transcriptome, as the process requires tissue dissociation and cell isolation. Spatial transcriptomics is a pivotal advancement in medical biotechnology, facilitating the identification of molecules such as RNA in their original spatial context within tissue sections at the single-cell level. This capability offers a substantial advantage over traditional single-cell sequencing techniques. Spatial transcriptomics offers valuable insights into a wide range of biomedical fields, including neurology, embryology, cancer research, immunology, and histology. This review highlights single-cell sequencing approaches, recent technological developments, associated challenges, various techniques for expression data analysis, and their applications in disciplines such as cancer research, microbiology, neuroscience, reproductive biology, and immunology. It highlights the critical role of single-cell sequencing tools in characterizing the dynamic nature of individual cells.

## Introduction

In recent years, significant progress in genomics, bioinformatics, biochemistry, material science, engineering, and computer-aided testing has led to the development of advanced high-throughput tools for gene profiling and sequencing from various biological specimens (Wu and Zhang, 2020). Modern technologies, such as RNA sequencing, now enable the simultaneous sequencing of multiple DNA fragments. As a result, researchers can gain a deep and comprehensive understanding of complex biological processes, including organism development, tissue regeneration, health and disease conditions, and cancer formation (Rego and Snyder, 2019; Zhang et al., 2021b).

These technological advancements, in turn, have driven the widespread adoption of sequencing-based tools to explore genomic heterogeneity and variations within biological systems. Among these tools, RNA sequencing (RNA-seq) stands out due to its exceptional precision. Specifically, it facilitates the discovery of new RNA species and enhances our understanding of transcriptomic changes (Choi and Kim, 2019; Li et al., 2020).

Building on this foundation, low-input RNA-sequencing methods have recently been adopted for single-cell analysis (Ding et al., 2020). Notably, single-cell RNA sequencing (scRNA-seq) provides a high-resolution view of individual cells within a population. By revealing cell-specific characteristics and changes that often remain hidden in bulk sequencing, this method enables the identification of rare cell subtypes and gene expression variations that would otherwise go unnoticed (Hwang et al., 2018).

The ability to analyze cells at the single-cell level is revolutionizing our understanding of organisms. For instance, it allows researchers to trace cell lineage and study tissue variability in detail (Teschendorff and Feinberg, 2021). By examining individual cells, we gain a unique perspective on the interactions between intrinsic cellular activities and external factors, such as environmental conditions or neighboring cell interactions, which influence cell fate. In clinical settings, single-cell analysis has proven invaluable in studying how rare “outlier” cells affect disease progression, drug resistance, and tumor relapse. Furthermore, this approach holds the potential to discover unknown microbial species or regulatory mechanisms crucial for biotechnology and medicine, especially given that many organisms cannot be cultured in laboratory conditions (Mangiola et al., 2023; Zhang et al., 2018).

As a result, the efficiency of single-cell RNA profiling has provided extraordinary insights into cellular heterogeneity across different organisms. Unlike bulk tissue analysis, which averages gene expression across many cells, single-cell sequencing uncovers the variability and probabilistic nature of gene expression. This approach allows for the sequencing of genetic material from individual cells, generating genomic, transcriptomic, or multi-omics data that highlight population heterogeneity and cell developmental relationships (Cha and Lee, 2020). However, traditional sequencing methods often fail to capture important cellular variations and struggle to analyze small cell populations. In contrast, single-cell sequencing excels at uncovering gene expression differences among individual cells and mapping cell trajectories. Notably, it was recognized as a major innovation in 2013 by Nature Methods. Despite its promise, early applications of single-cell sequencing were hindered by high costs and labor-intensive protocols (Evrony et al., 2021; Van den Berge et al., 2020).

Fortunately, advancements in instrumentation sensitivity and automation have addressed many of these challenges, making global single-cell analysis feasible. For example, high-throughput technologies now allow the parallel sequencing of numerous single cells, enabling the rapid generation of large datasets. Additionally, complementary techniques such as fluorescence and mass cytometry can identify expressed proteins. Meanwhile, messenger RNA (mRNA) can be studied using probe-based methods such as fluorescence *in situ* hybridization (FISH), real-time PCR (qRT-PCR), and microarrays, providing insights into the variability of gene expression across multiple genetic materials simultaneously (Liu et al., 2023; Mitra-Kaushik et al., 2021).

In conclusion, this overview highlights single-cell sequencing technologies, their development, challenges, and applications in fields such as cancer research, microbiology, neurology, reproductive health, and immunobiology. Nevertheless, despite their immense potential, single-cell RNA sequencing tools face significant challenges, including the need for specialized expertise and high costs, which limit their broader use in transcriptomic studies (Li et al., 2021; Lim et al., 2020).

### Single cell RNA sequencing

Single-cell RNA sequencing (scRNA-seq) analyzes the gene expression profiles of individual cells from both homogeneous and heterogeneous populations. To achieve this, the method isolates single cells, typically through encapsulation or flow cytometry, followed by the amplification and sequencing of RNA transcripts from each cell independently. Due to its high-resolution capabilities, scRNA-seq enables researchers to identify and characterize different cell types, states, and subpopulations (Stuart and Satija, 2019; Paik et al., 2020).

Currently, scRNA-seq is widely applied in life sciences research, particularly for comparing gene expression profiles between individual cells. Through this approach, researchers can discover and characterize novel or rare cell populations, refine our understanding of known cell types, and study critical biological processes such as cellular differentiation, lineage tracing, and developmental trajectories across various organisms. These insights provide a deeper understanding of the regulatory pathways that govern cellular fate decisions (Li et al., 2020; Ding et al., 2020).

## From bulk to single-cell transcriptomic dissection

Transcriptomic analysis at the single-cell level began about 20 years ago, pioneered by Norman Iscove, who employed polymerase chain reaction (PCR) for exponential amplification of single-cell cDNAs (Jena et al., 1996). Building on this foundation, James Eberwine advanced the field by developing a method to amplify cDNAs using T7 RNA polymerase-based transcription *in vitro* (Moll et al., 2004). These early innovations significantly enhanced the exploration of molecular systems involved in development and the intricate functioning of the vertebrate nervous system, where cellular diversity is particularly pronounced. In such cases, analyzing transcriptomes at the single-cell level, or even within specific regions like extended axons, has provided invaluable insights (Kulkarni et al., 2019).

**FIGURE 1:**
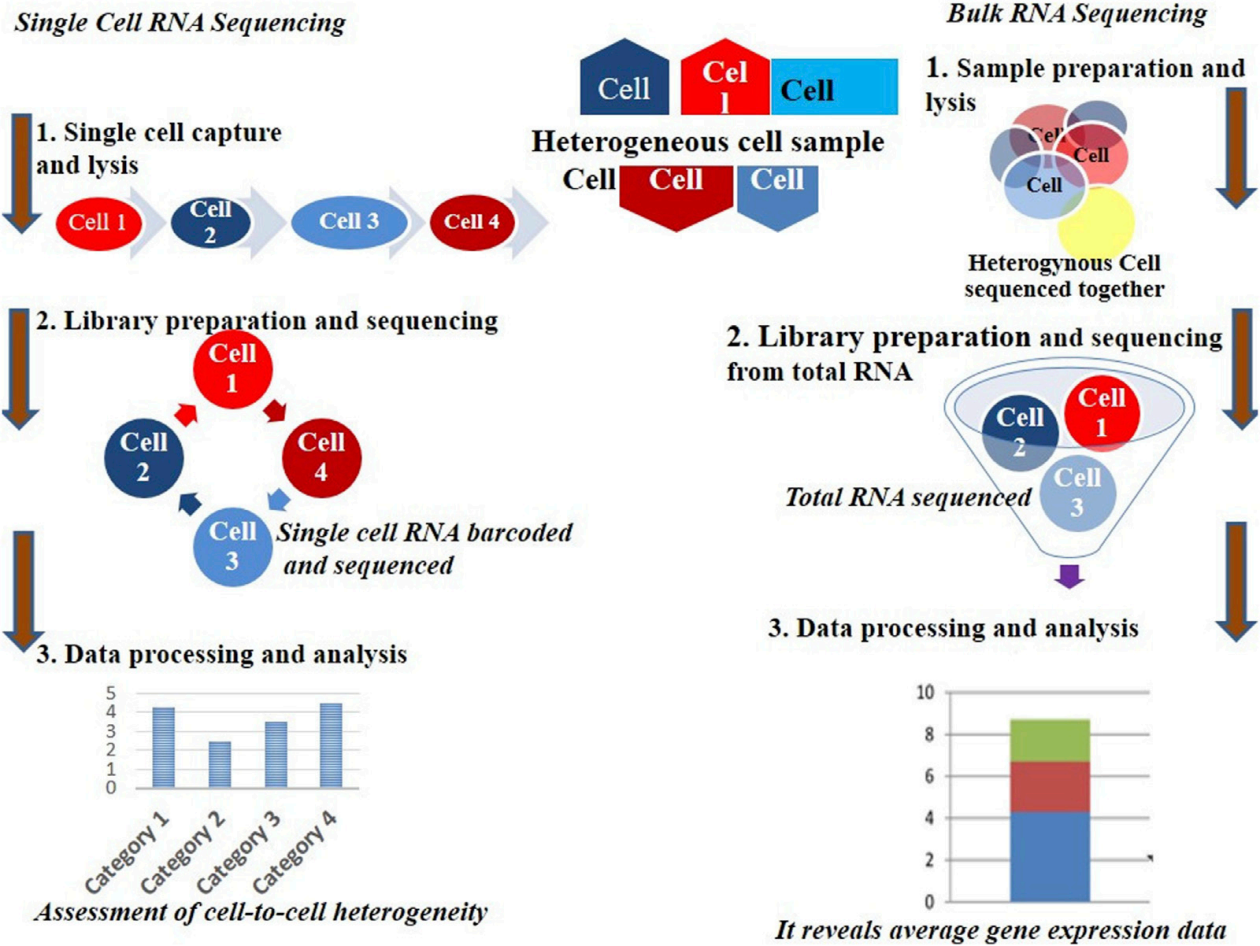
Mass and single cell RNA sequencing technique procedures.

The invention and mass production of high-density DNA microarray chips represented a major breakthrough in advancing individual cell microarray technologies. These cutting-edge chips enabled researchers to analyze gene expression with unprecedented precision, allowing for the study of individual cells rather than just bulk cell populations. Notably, recent studies have uncovered significant differences in the transcriptomes, the complete set of RNA molecules expressed of genetically identical cells, such as those from the same tissue or organism. This discovery revealed the remarkable complexity of cellular behavior and highlighted the critical importance of studying individual cells to gain a deeper understanding of biological processes. Furthermore, it exposed a key limitation of traditional population-level analyses, which often obscure cellular heterogeneity by averaging data, thereby masking the true dynamics and diversity of gene expression within cell populations (see Figure 1) (Gresham et al., 2008; Kurimoto et al., 2006).

To date, most transcriptomic analyses have been conducted at the population level, offering an average transcriptome derived from many cells. However, for specific conditions such as stem cells, tumor cells, or rare cell populations, obtaining sufficient material for analysis presents significant challenges. Additionally, traditional methods fail to capture the subtle but biologically significant variability that exists among seemingly identical cells (Fan et al., 2020). Although vertebrate cells are estimated to contain approximately 10⁵–10⁶ expressed mRNA molecules, the proportion of different transcriptomic groups within a population can vary greatly (Avila Cobos et al., 2020). This variability underscores the importance of single-cell RNA sequencing, which addresses these limitations. Figure 1 illustrates the workflow of mass *versus* single-cell RNA sequencing, emphasizing the distinct types of data generated by each method.

Single-cell RNA sequencing (scRNA-seq) has proven to be an invaluable tool for characterizing complex and diverse cell populations. By providing a detailed understanding of population composition, it enables the discovery of new subtypes and rare cell types. In the context of dynamic biological processes, scRNA-seq has been instrumental in reconstructing cell trajectories, offering insights into transient intermediate cell states and identifying key regulatory genes that drive these processes (Pokhilko et al., 2021; Li et al., 2021).

In addition to its role in trajectory analysis, scRNA-seq holds significant potential for studying probabilistic transcriptional bursting and unraveling gene regulatory systems. However, deriving hypotheses from scRNA-seq data presents computational challenges and difficulties in validation. To address these issues, system models inferred from the data must undergo rigorous testing and practical validation. Furthermore, mRNA expression levels can vary across a cell population due to either deterministic regulatory processes or random fluctuations, a phenomenon referred to as transcriptional noise. This variability is particularly important as it can have a significant impact on cell fate decisions (Luo et al., 2023; Edwards et al., 2023).

More broadly, scRNA-seq enables researchers to explore the distinct characteristics of individual cells within complex tissues and organ systems. This capability provides critical insights into how cellular subpopulations respond to environmental changes. Moreover, scRNA-seq has revealed the extent of transcriptomic variability, including both coding and non-coding RNAs, on a genome-wide scale. By offering a powerful approach to unravel time-dependent transcriptional networks during transitional processes or in response to external stimuli, scRNA-seq overcomes the limitations of population-level analyses, which often obscure such dynamics (Li et al., 2021). Table 1 compares bulk RNA sequencing with single-cell RNA sequencing, summarizing their key differences (Zhao et al., 2021).

**TABLE 1:** Key differences between bulk and single-cell RNA sequencing.

Bulk RNA seq	Single cell RNA seq
Measures the average gene expression across heterogeneous cells	Analysis gene expression profiles of individual cells
RNA from many different cell types are extracted	RNA from individual cells extracted
Millions of cells are pooled together into a single RNA sample	Each RNA transcripts investigated separately
Multiple elements (cells) are combined and sequenced together in a single run	Sequenced individually
Low resolution approach	Low resolution approach
Mask the important information’s available at small scale cellular level	Can show cellular heterogeneity and rear cell populations
Applied for gene expression profiling	Study cellular heterogeneity
Used for transcriptome annotation	Used for cell type identification and characterization
Typically less expensive and easier to perform	More technically challenging, requiring higher costs, advanced computational tools, and data analysis

## Progresses in single-cell RNA profiling techniques and innovations

**FIGURE 2:**
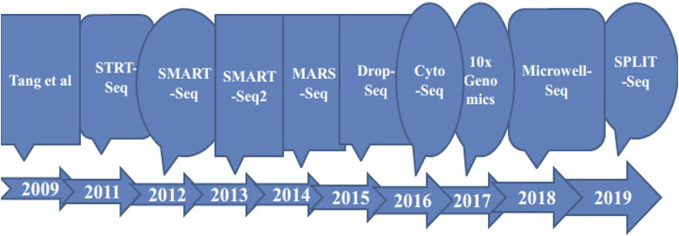
Timeline of some of improvements in single cell sequencing methods as a base for technology progressions to the next level.

Advances in life science research continue to accelerate, with scientists increasingly leveraging single-cell RNA sequencing tools to reduce the costs of dissecting cellular information. These advancements enable a more detailed exploration of the molecular behavior of individual cells, thereby enhancing our understanding of cellular biology (see Figure 2). Among these innovations is the Single-Cell Integrative Label Sequencing tool (SCI-seq), which simultaneously constructs multiple single-cell information libraries and analyzes cellular copy number heterogeneity. By significantly increasing the number of tissue cells that can be analyzed while reducing library preparation costs, SCI-seq offers substantial benefits for studying cellular diversity (Vitak et al., 2017; Chen et al., 2021).

Building on these developments, another study (Chen et al., 2017) introduced a novel single-cell whole-genome amplification technique capable of detecting copy number variations (CNVs) at the kilobase level. This method also facilitates the efficient identification of mutations associated with various diseases. Similarly, additional research (Guo et al., 2017) presented the scCOOL-seq technique, a parallel sequencing method for single cells. This tool provides simultaneous insights into genomic conditions, nuclear micro-environment organization, chromosome set duplications, and DNA methylation. Notably, scCOOL-seq can detect a range of genomic activities and DNA structures, including heritable epigenetic methylation, where a methyl group binds to specific gene locations (Clark et al., 2017). Furthermore, Topographic Single-Cell RNA Sequencing (TSCS) (Casasent et al., 2018) represents another groundbreaking approach. TSCS offers highly sensitive spatial information for individual cells, particularly cancer cells, allowing researchers to study their positional data and malignant behavior with remarkable precision.

Another notable technique highlights a highly efficient and minimally variable single-cell RNA sequencing approach that utilizes droplet-based microfluidics. This method isolates, amplifies, and barcodes the genetic material of individual cells, enabling a more comprehensive analysis of genetic material across diverse cell populations (De Jonghe et al., 2023). In addition, Microwell-seq, developed by another researcher (Han et al., 2018), is a sophisticated and cost-effective single-cell RNA sequencing method. It not only advances the study of various single-cell RNA tools but also significantly reduces examination costs by using multi-dimensional analysis encapsulated within oil droplets.

Similarly, SPLiT-seq, another cost-efficient tool based on the concept of barcoding, further reduces the expense of single-cell RNA transcriptomic sequencing to as low as one cent per cell. By lowering the cost barrier for single-cell analysis, SPLiT-seq makes this technology more accessible to a broader range of researchers (Rosenberg et al., 2018). Currently, no universally standardized single-cell sequencing techniques exist, so researchers typically select methods based on their specific study goals and available resources (Pullin and McCarthy, 2024).

Single-cell transcriptome sequencing tools can generally be categorized into various types based on the portion of the transcript they cover. These categories include full-length transcriptome sequencing methods, such as MATQ-seq (Sheng et al., 2017), SMART-seq2 (Picelli et al., 2013), ICELL8 (Goldstein et al., 2017), and SUPeR-seq (Fan et al., 2015), as well as 5′-end transcriptome sequencing techniques, exemplified by STRT-seq (Islam et al., 2011). Additionally, 3′-end transcriptome sequencing techniques, such as Chromium (10X Genomics) (Zheng et al., 2017) (10X Genomics), Fluidigm C1 (DeLaughter, 2018), Drop-seq (Macosko et al., 2015), and inDrop (Klein et al., 2015), offer distinct benefits depending on the research objectives.

Full-length transcriptomic sequencing techniques in single-cell RNA sequencing (scRNA-seq) offer a unique advantage by capturing complete RNA molecules, including their 5′ and 3′ ends. This holistic approach allows for the simultaneous analysis of both transcriptomic and genotypic data at the single-cell level. By detecting full-length isoforms and single nucleotide variants (SNVs), these methods enable researchers to directly correlate genetic variations with their effects on gene expression. This is particularly useful in studies examining the effects of pathogenic genetic variants, as it provides essential insights into how specific mutations affect gene expression, splicing, and cellular functions. These integrated results are crucial for understanding disease mechanisms and informing the development of targeted therapies (Chen G. et al., 2019; Choi et al., 2020).

However, full-length transcriptomic sequencing methods also face several challenges, including lower accuracy, slower processing speeds, and higher costs (Byrne et al., 2019). While alternative approaches that focus solely on the 5′ or 3′ ends of transcripts can mitigate some of these issues, they restrict the ability to investigate allele-specific gene expression and alternative splicing. Many full-length sequencing techniques, such as MARS-seq, depend on flow-activated cell sorting (FACS), which requires a large starting sample volume. This limitation makes these methods impractical for small sample sizes, such as those from fine-needle aspiration. Additionally, FACS necessitates the use of antibody beads for cell sorting, which can hinder the classification of rare cell subtypes. Thus, each method comes with trade-offs that influence data depth, quality, and the subsequent biological and numerical interpretations (Keren-Shaul et al., 2019; Li et al., 2012).

Unlike mass sequencing, single-cell RNA sequencing is not a “one-size-fits-all” approach. The depth of analysis varies depending on the protocols used, the types of cells being studied, isolation techniques, sequencing methods, and the stringency applied during library preparation (Pedersen and Olsen, 2020). The process begins with isolating individual cells from biological samples, but this step poses a significant challenge due to the need for accurate cell capture. To improve the precision of transcriptome expression analysis, highly sensitive isolation techniques and skilled personnel are essential. Several methods are available for isolating single cells from biological samples, including limiting dilution, FACS, micromanipulation, laser capture microdissection (LCM), and microfluidics (Gautam et al., 2021).

The limiting dilution method uses pipettes to isolate targeted cells from mixed populations by diluting the sample. While simple, this technique is less productive compared to other methods. In contrast, microdissection or microinjection is typically employed for isolating cells from very small quantities, such as early embryos or uncultured microorganisms. Although effective for these small samples, these methods are labor-intensive and have low throughput (Fuadiyah et al., 2022).

Fluorescence-Activated Cell Sorting (FACS) is widely used for selecting individual cells, but it requires processing large volumes (typically >10,000 cells) in suspension. Known for its efficiency, FACS is, however, hindered by slow sample processing (Sutermaster and Darling, 2019). On the other hand, Laser Capture Micro-Dissection (LCM) is a sophisticated technique that uses a laser, guided by a computer, to select individual cells from solid tissue specimens. While offering rapid processing, LCM requires optical microscopy for identifying single cells within complex tissues, as well as specialized expertise (Griesser et al., 2020).

Emerging as a popular technique, microfluidics offers minimal specimen usage, precise fluid control, and low costs. This method is advantageous for isolation efficiency and clarity in targeting cells. Ultimately, each method has its own strengths and weaknesses, making them suitable for different applications in single-cell isolation (Lamanna et al., 2020).

Different single-cell RNA sequencing (scRNA-seq) methods offer distinct advantages and limitations, and numerous analyses have compared these techniques extensively. For example, Smart-seq2 is recognized for its ability to identify a larger number of expressed genes compared to other scRNA-seq tools, including CEL-seq2 (Hashimshony et al., 2016), MARS-seq (Jaitin et al., 2014), Smart-seq (Ramsköld et al., 2012), and Drop-seq (Ziegenhain et al., 2017). However, recent work by Sheng et al. has shown that MATQ-seq, another full-length transcriptome sequencing method, can outperform Smart-seq2 in detecting genes with low expression levels (Sheng et al., 2017).

Full-length single-cell RNA sequencing techniques, such as MATQ-seq, are particularly valuable for investigating isoform usage, allelic expression, and RNA editing due to their comprehensive coverage of the transcriptome. These methods offer distinct advantages over 3′-end or 5′-end sequencing approaches, especially when studying lowly expressed transcripts (Hagemann-Jensen et al., 2020). On the other hand, droplet-based tools like Drop-seq (Macosko et al., 2015), InDrop (Klein et al., 2015), and Chromium (Zheng et al., 2017) excel in providing high-throughput parallel sequencing at a lower cost per cell. These techniques are ideal for analyzing large numbers of cells, making them particularly effective in detecting sub-populations within complex tissues or cancer specimens. Additionally, some single-cell RNA sequencing methods, such as SUPeR-seq (Fan et al., 2015) and MATQ-seq (Sheng et al., 2017), can capture both polyA+ and polyA− RNAs. This ability is crucial for sequencing long non-coding RNAs and circular RNAs, further enhancing the utility of these advanced techniques.

To assess technical differences between various cell types and spike-ins, such as External RNA Controls Consortium (2005), Unique Molecular Identifiers (UMIs) are commonly employed in single-cell RNA sequencing techniques. RNA spike-ins transcripts with known sequences and quantities—are used to calibrate RNA hybridization assays like RNA sequencing. UMIs improve the accuracy of molecular counts by distinguishing between unique RNA molecules (Ziegenhain et al., 2022).

While spike-ins are used in methods such as Smart-seq2 and SUPeR-seq, they are not compatible with droplet-based techniques. Conversely, UMIs are predominantly employed in 3′-end sequencing methods like Drop-seq (Macosko et al., 2015), InDrop (Klein et al., 2015), and MARS-seq (Jaitin et al., 2014). Therefore, researchers can choose the most suitable single-cell RNA sequencing method based on practical factors, such as the number of cells to be sequenced and cost considerations.

### Current progresses in single-cell RNA sequencing tools

The investigation of cells dates back to the 16th century, beginning with early observations through rudimentary microscopes invented by Zacharias Janssen and Hans Lippershey (Hawaldar, 2017). In the 17th century, pioneers like Robert Hooke and Anton van Leeuwenhoek observed the first living cells. However, it took nearly two centuries before cells were fully understood as both structural and functional units of life. Early research aimed to enhance the perception and study of cells within complex multicellular networks (Mazzarello, 1999).

The development of single-cell RNA sequencing (scRNA-seq) began with the sequencing of transcripts from single blastomeres and oocytes (Tang et al., 2009). This breakthrough led to the introduction of new methods for scaling up cell quantities and improving RNA sequencing techniques (see Table 2). Over time, significant reductions in costs and advancements in automation have greatly enhanced scRNA-seq technologies. Despite these improvements, the core concept of single-cell RNA sequencing analyzing gene expression at the single-cell level has remained consistent (Wang S. et al., 2023).

**TABLE 2:** Advancements in single-cell RNA sequencing technologies.

Methods	Applications in single cell RNA sequencing
SCI-seq	Can simultaneously construct multiple single cell information libraries and analysis heterogeneity in body cell copy number
scCOOL-seq	Sequencing single cells by parallel method and analysis of methylated DNA
TSCS	Enabling precise spatial positional data for individual cells
Microwell-seq	Significantly reduce the cost of examination by one cent
SMART-seq2	Enable entire length transcriptome sequencing mechanisms
Drop-seq and Chromium	Can give huge parallel sequencing of single cells
SPLiT-Seq	Analyzing single cells in tissues where isolation is challenging
InDrops	Identifying transcriptional changes in response to stimuli and high-throughput gene expression profiling
sci-RNA-seq	Mapping developmental trajectories
CITE-Seq	Simultaneous profiling of gene and protein expression
ATAC-Seq + scRNA-Seq	Investigating the interplay between chromatin state and transcriptional activity
Seq-Well	Profiling rare cell types or small tissue samples and studying cellular dynamics in resource-limited settings
10x Genomics Chromium	Mapping cellular heterogeneity in tissues and analyzing immune responses in cancer or infectious diseases

Single-cell investigations in humans have profoundly advanced our understanding of developmental processes, biological activity, aging, and disease characterization, including tumor development (Puram et al., 2017). However, creating universal standards for single-cell RNA transcript analysis continues to present challenges. Each method requires careful decision-making to ensure meaningful results. This includes selecting the appropriate specimen types, determining cell quantities and preparation methods, choosing suitable single-cell RNA sequencing techniques and protocols, and designing effective computational analysis frameworks. Ultimately, successful single-cell RNA transcriptomic analysis, yielding interpretable data and relevant scientific insights, depends on well-defined experimental protocols (Lafzi et al., 2018; Zhang Z. et al., 2021).

### Single-cell isolation process and library preparation

Single-cell isolation and library preparation are critical steps in single-cell RNA sequencing (scRNA-seq), enabling the study of gene expression at the individual cell level. The first step is isolating individual cells from tissues or heterogeneous populations, which is essential for capturing high-quality single cells while preserving their genomic and biochemical integrity. This isolation process allows for a detailed analysis of specific genomic and molecular operations. There are several methods to achieve this, including Fluorescence-Activated Cell Sorting (FACS), Microfluidic Devices (e.g., Drop-Seq or 10x Genomics), Laser Capture Microdissection (LCM), Limiting Dilution, Magnetic-Activated Cell Sorting (MACS), and manual pipetting (Arsenio, 2020; Sant et al., 2023).

The methods used for isolating single cells and capturing their profiles vary depending on the organism, tissue, or cell type being studied. Cells can be isolated by selecting whole cells, isolating specific cell nuclei or organelles, or targeting cells expressing specific marker proteins. Each isolated cell’s transcriptome is then uniquely barcoded before its RNA is converted into complementary DNA (cDNA) (Sant et al., 2023). However, scRNA-seq methods are not without challenges, including the issue of 'artificial transcriptional stress responses.’ These responses occur when the cell separation process inadvertently activates stress-related genes, leading to synthetic alterations in the cell’s transcriptional profile. For instance, protease-based cell separation at 37°C has been shown to induce stress-related gene expression, potentially resulting in inaccuracies in cell type identification (van den Brink et al., 2017).

Once the cells are isolated, the next critical step is library preparation, which converts RNA into a form suitable for sequencing. The isolated cells are lysed to release their contents, including RNA. mRNA is then captured, and subsequently converted into cDNA. Full-length cDNA is synthesized from mRNA transcripts using reverse transcriptase with terminal transferase activity. This enzyme, in combination with a second “template-switch” primer, produces cDNAs with two universal priming sequences. The cDNA is then fragmented and ligated to sequencing adapters, making it compatible with high-throughput sequencing platforms. The adapters used are specific to the sequencing technology, such as Illumina or PacBio. Once barcoded cDNAs from each isolated cell or nucleus are generated, they can be sequenced using various high-throughput sequencing techniques, yielding reads for downstream bioinformatics analysis (Trombetta et al., 2014).

### Single-cell transcriptomic sequence data analysis

Analyzing single-cell RNA sequencing (scRNA-seq) data enables the discovery of novel cellular subpopulations, pathways, and mechanisms, thereby transforming biomedical research with advances in computational tools and techniques (Li et al., 2020). As scRNA-seq techniques continue to evolve, particularly in the context of clinical specimens, the analysis of resulting large datasets has become increasingly challenging. However, this step is crucial for fully leveraging these advanced techniques in life sciences and clinical studies. The landscape of scRNA-seq data analysis is rich with tools and methods designed to handle the complexity and scale of single-cell data. Notably, nearly 1,000 bioinformatics tools have been developed and made available as of 28 May 2021 (Hwang et al., 2018). Among these, several software tools, including Seurat, Scanpy, Monocle, and CellRanger, are widely used to facilitate tasks such as clustering, dimensionality reduction, and trajectory analysis. For visualization, methods like UMAP, t-SNE, and violin plots are extensively employed to display data and highlight gene expression patterns. Additionally, data repositories such as the Human Cell Atlas and Single Cell Portal provide valuable reference datasets for cell-type identification and cross-study comparisons (Zappia et al., 2018; Holland et al., 2020).

The analysis of single-cell transcriptomic sequencing data involves several critical steps. First, data preprocessing ensures quality by filtering out low-quality cells and normalizing read counts to correct for technical variability. Following this, highly variable genes are selected for further analysis. To simplify data visualization while preserving essential features, dimensionality reduction techniques like PCA, t-SNE, or UMAP are applied. Subsequently, clustering algorithms identify groups of cells with similar expression patterns, and these clusters are annotated using marker genes to determine cell types. Differential gene expression analysis is then performed to highlight genes with significant changes between populations. Furthermore, for studying dynamic processes, trajectory or pseudotime analysis reconstructs cellular differentiation paths. Finally, integrating data from multiple experiments ensures consistency and broader insights while addressing challenges such as batch effects and dropout events (Wu and Zhang, 2020; Shi, 2021).

In scRNA-seq data analysis, several challenges must be addressed. For example, batch effects, arising from technical variability across experiments, can be mitigated using integration tools to ensure consistent results. Additionally, dropout events, where some RNA molecules are not captured, contribute to data sparsity and require specialized handling to avoid bias. Moreover, the computational complexity of analyzing large datasets often demands high-performance computing resources to manage processing requirements efficiently. Together, these approaches are crucial for ensuring the reliability and efficiency of scRNA-seq data analysis (Kiselev et al., 2019; Kharchenko, 2021).

During the preparation of single-cell mixtures, issues such as cell death, membrane damage, or multi-cellular adhesion can arise due to natural conditions and experimental challenges. To mitigate the impact of low-quality cells on gene expression data, quality control (QC) measures are essential. Techniques like Seurat (Stuart et al., 2019), scran (Lun et al., 2016), and scanpy (Wolf et al., 2018) are commonly used for this purpose. Seurat has become an extensively utilized R package designed for the analysis and interpretation of single-cell RNA sequencing (scRNA-seq) data. It provides a variety of functionalities for quality assessment, preprocessing, and dimensionality reduction. For instance, it enables filtering cells based on parameters like mitochondrial gene content and normalizes datasets to mitigate technical inconsistencies. The package facilitates the clustering of cells into specific subpopulations by examining gene expression profiles and supports differential expression analysis between these groups. Furthermore, Seurat allows the integration of diverse data types, such as gene expression and protein markers, while offering visualization methods like t-SNE and UMAP to uncover cellular diversity. It also incorporates advanced tools for trajectory analysis, spatial transcriptomics, and gene set enrichment analysis, positioning itself as an all-in-one solution for scRNA-seq data processing (Huang et al., 2021). For instance, Seurat assesses several metrics to determine whether a cell should be retained, including the number of genes, the number of unique molecular identifiers (UMIs), the proportion of mitochondrial genes, and the ratio of ribosomal protein genes (Pereira et al., 2021).

Notably, there is no one-size-fits-all filter threshold, as it depends on the cell types and tissues being analyzed. For example, some studies suggest thresholds such as a maximum of 100 or a minimum of 6,000 expressed genes, a maximum of 200 UMIs, and a minimum of 10% mitochondrial genes (Lambrechts et al., 2018). Another study recommended thresholds of 200–2,500 genes per cell, 300–15,000 UMIs per cell, and less than 10% mitochondrial genes (Fan et al., 2019).

In scRNA-seq analysis, each cell is treated as an individual sample. However, raw expression data often cannot be used directly due to systemic biases and technical noise, such as variations in sequencing depth and transcript capture efficiency. Thus, normalization is essential to address these issues and ensure comparability across cells. An evaluation of seven normalization techniques, including BASiCS, GRM, Linnorm, SAMstrt, SCnorm, scran, and Simple Norm, highlights the importance of proper normalization (Lytal et al., 2020; Hafemeister and Satija, 2019).

Typically, scRNA-seq datasets include thousands of cells and millions of genes. Many of these genes are housekeeping genes with little variation, which may obscure meaningful biological information (Choudhary and Satija, 2022). To overcome this, identifying highly variable genes (HVGs) with significant cell-to-cell expression differences is critical for detecting biological stimuli and reducing computational burden. The quality of HVGs significantly affects cell grouping accuracy. A study evaluating seven techniques for identifying HVGs, including BASiCS, Brennecke, scLVM, scran, scVEGs, and Seurat, found significant differences in performance and operational times. For example, scran identified a reliable number of HVGs with good performance, while Brennecke showed stable results across various datasets. Moreover, scran and Seurat performed well in certain datasets, whereas BASiCS and scLVM_LogVar were noted for their stability compared to other methods (Yip et al., 2019; Miao et al., 2020).

#### Data preprocessing

Proper preprocessing is essential to ensure the quality and accuracy of scRNA-seq data. The process begins with stringent quality control to exclude low-quality cells, often identified by irregular metrics such as elevated mitochondrial gene expression or reduced unique molecular identifiers (UMIs). Next, normalization is performed to adjust raw read counts, compensating for differences in sequencing depth and technical biases, thus enabling meaningful comparisons between cells. This step is critical for achieving consistent gene expression measurements by scaling raw data to a standard baseline. Common normalization methods include library size scaling, log transformation, and advanced approaches like SCTransform or scran. Accurate normalization is vital for subsequent analyses, such as clustering, differential gene expression, and trajectory inference, as it reduces technical noise and highlights genuine biological differences (Hafemeister and Satija, 2019). Next, feature selection is performed to identify highly variable genes that capture the most biologically relevant variations in the dataset, thus setting the stage for downstream analyses (Choudhary and Satija, 2022).

Dimensionality reduction follows preprocessing as an essential step to simplify the complexity of scRNA-seq datasets. Techniques such as Principal Component Analysis (PCA), t-SNE (t-Distributed Stochastic Neighbor Embedding), and UMAP (Uniform Manifold Approximation and Projection) are commonly employed to transform high-dimensional data into fewer dimensions while preserving essential biological information. By doing so, these methods facilitate the visualization and interpretation of data, revealing patterns and relationships between cells, such as the clustering of similar cell populations, in a more comprehensible two- or three-dimensional space (Townes et al., 2019; Raimundo et al., 2020).

Building on dimensionality reduction, clustering group’s cells with similar gene expression profiles to uncover distinct cell populations. To achieve this, algorithms like k-means or Louvain are commonly used to identify clusters based on shared patterns in the data. After clustering, cell-type annotation assigns biological identities to each group by comparing their gene expression signatures with known marker genes or reference datasets. This step is fundamental for understanding the functional roles of the identified cell populations within their biological context (Chen L. et al., 2020; Hozumi et al., 2023).

In many studies, integrating datasets from multiple samples or experiments is a critical step to gain a comprehensive understanding of biological variability across different conditions or subjects. Tools like Seurat and Harmony are often utilized for merging scRNA-seq data, correcting batch effects, and addressing technical discrepancies between datasets. This integration allows for more robust analysis and facilitates clearer comparisons of cellular features, ultimately improving the generalizability of findings across diverse experimental settings (Butler et al., 2018; Ryu et al., 2023).

The initial processing of unprocessed single-cell transcriptome sequencing datasets often begins with handling data in FASTQ and BCL formats, depending on the source and sequencing platform. For standard quality control, data must first be in FASTQ format (Andrews et al., 2021). When dealing with BCL data, conversion is required, and tools such as the cellranger mkfastq pipeline, which leverages the banded bcl2fastq program, are commonly employed. This process requires a CSV matrix dataset with at least three columns: lane, sample, and index alongside the path to the BCL extracts. After conversion to FASTQ format, tools like FastQC can assess the quality of raw single-cell RNA sequencing data. This step ensures data integrity and identifies potential issues early in the analysis pipeline, thereby supporting reliable downstream analyses (Brown et al., 2017; Andrews et al., 2021).

#### Exploratory analysis

To uncover functional errors and biological significance within cell populations, conducting functional enrichment analyses on differentially expressed genes is essential. Among the widely used methods, Gene Ontology (GO) and Kyoto Encyclopedia of Genes and Genomes (KEGG) pathway analysis stand out as effective tools for interpreting single-cell datasets. Numerous techniques for functional enrichment have been developed, with Huang et al. providing a comprehensive evaluation of 68 methods, highlighting their respective strengths and limitations (Fang et al., 2021).

Building upon functional enrichment, trajectory and pseudotime analysis reconstructs dynamic biological processes, such as cell differentiation or disease progression, by arranging cells along a continuum based on their gene expression profiles. Algorithms like Monocle and Slingshot are instrumental in inferring the order and relationships between cells, offering insights into temporal changes or developmental pathways. This approach is particularly valuable for understanding transitions between cell states and identifying key regulatory genes driving these processes (Cheng et al., 2023). For pathway-based investigations, Gene Set Variation Analysis (GSVA) is especially valuable. GSVA evaluates enrichment outcomes across various signaling pathways, thereby elucidating the sources of morphological variations. When combined with KEGG pathway analysis, GSVA provides physiologically informative results that enhance our understanding of cellular dynamics (Zhong et al., 2022).

To identify enriched transcription factors within each cell group, the SCENIC (Single-Cell Regulatory Network Inference and Clustering) technique is a widely used method. SCENIC identifies transcription factors by detecting enrichment of TF motifs and their relationships with target genes (Van de Sande et al., 2020). While SCENIC can be implemented in both R and Python, pySCENIC is recommended for large datasets due to its superior performance. The latest version of SCENIC supports analysis for *Homo sapiens*, *Mus musculus*, and *Drosophila melanogaster*, with the flexibility to construct custom databases for other species. Despite its versatility, SCENIC has faced criticism for its limited ability to capture dynamic changes in gene regulation across different cell types (Van de Sande et al., 2020). An alternative to SCENIC is IRIS3, a cell-class-specific regulon reasoning server. IRIS3 is particularly appreciated for its user-friendly web interface, making it accessible to users with limited programming experience. However, there remains room for improvement in its reliability and efficiency, which could enhance its adoption and impact in single-cell RNA sequencing studies (Ma et al., 2020).

Overall, scRNA-seq data analysis has revolutionized our understanding of cellular biology by enabling the discovery of novel cell types, developmental pathways, and gene regulatory mechanisms. Its integration with other data types and application in disease research, particularly in cancer and cardiovascular diseases, underscores its transformative impact on biomedical research (Hwang et al., 2018; Zhang et al., 2021b).

## Spatial single-cell RNA sequencing

The spatial context of cells within multicellular organisms is vital for understanding their functions and interactions. For instance, stem cell differentiation during development is shaped by cell-to-cell interactions and signaling, which are regulated by the cells’ relative positions within the embryo. This spatial localization influences transcription factor expression and contributes to cellular organization and functionality (Bingham et al., 2020). To explore such spatial relationships, spatial transcriptomics has emerged as a powerful collection of technologies. Unlike single-cell RNA sequencing (scRNA-seq), which isolates cells from their native environments, spatial transcriptomics retains spatial relationships, offering valuable insights into how gene expression is influenced by cellular microenvironments and tissue architecture (Rao et al., 2021; Liao et al., 2021).

While scRNA-seq provides high-resolution gene expression data, it loses the spatial context of tissue architecture during cell dissociation. This disruption complicates the study of tissue-specific functions, cell-to-cell interactions, and spatially defined disease progression. Consequently, scRNA-seq alone may fail to offer a complete picture of cellular interactions or the influence of spatial localization on gene expression within tissues. To address this limitation, spatial transcriptomics preserves tissue organization while enabling gene expression analysis. This allows researchers to examine the spatial distribution of cells and their interactions in a more natural and meaningful context (Longo et al., 2021; Ahmed et al., 2022).

Building on this concept, Location-Based Transcriptomics maps the spatial distribution of RNA molecules within tissue sections or individual cells. This technique enables the identification of specific transcript expressions within intact tissue, thereby providing a detailed spatial context at the cellular or sub-cellular level. Consequently, it enhances our understanding of tissue heterogeneity, cellular diversity, and the spatial distribution of gene expression, offering deeper insights into tissue function and sub-cellular RNA localization (Longo et al., 2021).

Despite its advantages, spatial transcriptomics has notable limitations compared to scRNA-seq. One major drawback is its lower resolution, as many spatial techniques analyze gene expression at the tissue region or spot level, encompassing multiple cells rather than individual ones. For example, methods like Visium capture gene expression from spatially barcoded regions of tissue, each containing multiple cells, thereby producing aggregated data rather than single-cell resolution. This aggregation can obscure cellular heterogeneity and complicate the identification of individual cell types or interactions in densely packed tissues. Moreover, spatial transcriptomics often focuses on a limited set of genes due to technical constraints, whereas scRNA-seq provides genome-wide expression profiles (Longo et al., 2021; Lee et al., 2022).

Additionally, spatial transcriptomics faces challenges related to sensitivity, as low-abundance transcripts can be difficult to detect. The experimental complexity and resource demands, including specialized equipment and meticulous tissue handling, further contribute to the limitations of this technology. Interpreting the data can also be challenging, as the blending of gene expression from multiple cells within a spatial spot masks finer cellular details. In densely packed tissues like the brain or tumors, spatial transcriptomics may fail to resolve intricate cellular interactions, making it less effective in such contexts. Despite these challenges, spatial transcriptomics complements scRNA-seq by preserving tissue context, albeit with trade-offs in resolution, sensitivity, and gene coverage (Cable et al., 2022; Li et al., 2023).

To categorize spatial transcriptomics techniques, they can be divided into two main approaches: Next-Generation Sequencing (NGS)-based methods and Imaging-based methods. Each has unique principles and applications, offering complementary ways to spatially resolve gene expression (Rao et al., 2021). NGS-based spatial transcriptomics focuses on RNA transcript localization within tissue sections before high-throughput sequencing. This involves placing tissue samples on slides embedded with spatially barcoded capture probes, which bind RNA molecules, preserving their spatial coordinates. Sequencing then generates comprehensive transcriptomic data mapped back to the original spatial locations. Prominent examples like 10x Genomics Visium and Slide-seq exemplify NGS-based methods. However, these methods often lack single-cell resolution, limiting their ability to capture fine cellular heterogeneity (Larsson et al., 2021; Liu et al., 2020).

Imaging-based spatial transcriptomics utilizes approaches like *in situ* sequencing (ISS) and *in situ* hybridization (ISH) to examine RNA directly within tissue sections. These techniques amplify RNA molecules and identify target genes through sequencing or hybridization. For example, RNAscope employs fluorescent probes to detect specific RNA targets with exceptional spatial resolution. Similarly, methods such as MERFISH (Multiplexed Error-Robust Fluorescence *In Situ* Hybridization) and seqFISH (Sequential Fluorescence *In Situ* Hybridization) enable visualization of the spatial distribution of hundreds or even thousands of transcripts simultaneously. The key advantage of these imaging-based methods is their ability to achieve near single-cell resolution while maintaining the structural integrity of the tissue. However, they are limited by their focus on predefined gene sets rather than providing a comprehensive view of the entire transcriptome (Gyllborg et al., 2020; Petukhov et al., 2022).

Spatial transcriptomics has diverse applications across biomedical fields. In neurology, it maps gene expression in brain tissues, revealing insights into neuronal organization, connectivity, and function. This has been particularly valuable in understanding disorders like Alzheimer’s disease, Parkinson’s disease, and autism, where spatially defined gene expression changes play significant roles in disease mechanisms (Ya et al., 2023).

In embryology, spatial transcriptomics gives significant insights into gene expression models during development, helping researchers to explore processes like cell differentiation, tissue morphogenesis, and organ formation. Similarly, in cancer research, this technology uncovers tumor heterogeneity, maps the tumor microenvironment, and investigates interactions between cancer cells and their stromal or immune counterparts. These insights improve our understanding of cancer biology and inform better diagnostic and therapeutic strategies (Rao et al., 2021; Yu et al., 2022).

In the context of cancer, spatial transcriptomics enables for a detailed analysis of tumor heterogeneity by identifying particular gene expression patterns across different tumor regions. This helps to elucidate why certain tumor areas are more aggressive or treatment-resistant. Moreover, it facilitates the investigations of interactions between cancer cells and their microenvironment, including stromal cells, immune cells, and extracellular matrix components. By preserving tissue architecture, spatial transcriptomics maps critical cellular processes including angiogenesis, immune evasion, and therapy resistance within the tumor’s spatial context. It also aids in detecting spatially distinct biomarkers, enhancing cancer diagnosis, prognosis, and the assessment of therapeutic impacts on gene expression and cell interactions (Hu et al., 2022; Wang N. et al., 2021).

By preserving tissue architecture, spatial transcriptomics allows the mapping of key cellular processes like angiogenesis, immune evasion, and therapy resistance within the tumor’s spatial context. It also supports the identification of spatially distinct biomarkers that can improve cancer diagnosis and prognosis. Additionally, this technology aids in assessing the impact of therapies at the molecular level by visualizing how treatments affect gene expression and cell interactions within specific tumor regions. These applications make spatial transcriptomics a powerful tool for advancing precision oncology and developing more effective cancer treatments (Li et al., 2022; Yu et al., 2022).

Overall, spatial transcriptomics integrates spatial information with transcriptomic data, providing a comprehensive understanding of cellular functions within native tissue architecture. By preserving spatial context, this technology enables the study of complex biological processes, including cellular differentiation, tissue development, and microenvironment interactions. Its ability to correlate gene expression with structural features makes it a transformative tool for advancing basic research and translational medicine (Rao et al., 2021; Liao et al., 2021).

## Single-cell sequencing applications in the field of biomedical research

Single-cell RNA transcriptomic investigation has emerged as a transformative tool in modern science, celebrated for its precision and efficiency in unraveling cellular complexity across various fields, including human, animal, and plant research. By enabling the detailed analysis of individual cells, this technology offers unparalleled insights into rare cell types, their unique gene expression profiles, and the intricate interactions within cellular ecosystems. These capabilities have revolutionized our understanding of biological systems, offering new perspectives on cellular configurations and their roles in health and disease. For instance, the ability to identify elusive subpopulations of cells and uncover molecular mechanisms driving pathological processes has opened new research frontiers, facilitating advancements in personalized medicine, developmental biology, and agricultural science. As its applications continue to expand, single-cell transcriptomics provides critical tools for addressing complex biological challenges (Shojaee et al., 2021).

Although initially developed for the study of animal and human cells, single-cell RNA sequencing is now gaining traction in plant science. However, its application in this domain remains relatively novel and underexplored. Transitioning to plant systems presents unique challenges, such as practical limitations and a less comprehensive understanding of plant cell diversity compared to animal cells. Despite these obstacles, researchers have made significant progress, particularly with *Arabidopsis thaliana*, a model organism extensively used in molecular genomics. Arabidopsis is favored for its manageable cell count, well-characterized gene markers, and robust cell isolation methods. For example, enzymatic degradation of the cell wall has emerged as an effective technique for isolating plant cells, enabling the detailed study of their gene expression profiles. These advances in single-cell transcriptomics are unlocking unprecedented insights into plant biology, with applications ranging from developmental studies and stress responses to crop improvement (Bawa et al., 2022; Sun et al., 2024).

Single-cell transcriptomic analysis provides an in-depth understanding of cellular processes, revealing how individual cells interact with their surroundings and respond to local factors and neighboring cells. This detailed approach is particularly valuable in clinical diagnostics, as studying the behavior of unique “outlier” cells can yield critical insights into disease progression, antimicrobial resistance, and tumor dynamics. Since atypical cells often play a central role in driving pathological conditions, their investigation is vital for creating targeted therapies. The emergence of advanced single-cell RNA sequencing technologies has transformed this field, allowing researchers to uncover the molecular basis of cellular behavior with unparalleled accuracy. Consequently, single-cell analysis has become essential for deciphering complex disease mechanisms, refining treatment strategies, and advancing drug discovery. By linking molecular insights to clinical applications, single-cell technologies are setting the stage for more personalized and effective therapeutic solutions (Algabri et al., 2022; Mohammadi et al., 2019).

Tissues and organs are composed of highly organized and functionally diverse groups of cells, with variations influenced by physiological changes, differentiation pathways, and spatial contexts. While these microenvironments typically remain stable under normal conditions, they can be disrupted in extreme scenarios, such as tumor formation. To better understand tumors—including their development, cell origins, growth, malignancy, and treatment responses—it is essential to analyze the tumor microenvironment, with a particular focus on immunological and stromal components (Hinshaw and Shevde, 2019).

Single-cell transcriptomic sequencing enables detailed analysis of both healthy and tumor cells across different stages of tumor development. This capability facilitates accurate comparisons and assessments of treatment efficacy, ultimately leading to more effective therapeutic strategies. Initially, single-cell RNA sequencing (scRNA-seq) focused on analyzing distinct tissue regions and cell types, generating extensive datasets for deeper insights (Lei et al., 2021). Furthermore, single-cell RNA sequencing data can be utilized to infer gene regulatory networks (GRNs) by clustering genes into co-regulated “modules” based on similarities in their expression patterns. This approach enhances our understanding of the regulatory relationships and interactions among genes, further advancing the field of tumor biology (Dautle et al., 2023).

### Applications in cancer research

Single-cell RNA sequencing (scRNA-seq) has significantly transformed cancer research by offering detailed insights into tumor heterogeneity, the tumor microenvironment, malignancy, and resistance to treatments. Unlike traditional bulk sequencing, which averages data across all cells, scRNA-seq examines individual cancer cells, revealing genetic and phenotypic variations that may be overlooked by conventional methods. This high-resolution technology uncovers differences between cancer cells, including rare subpopulations that drive tumor growth and resist therapies. By focusing on single cells, scRNA-seq provides a clearer understanding of oncogenic pathways, gene expression, and cellular behaviors, offering valuable information for cancer biology research (Zhang et al., 2021b; Hong et al., 2020).

In addition to deciphering tumor cell diversity, scRNA-seq offers a comprehensive view of the tumor microenvironment, uncovering interactions between cancer cells and their surrounding non-cancerous environments, including immune cells, stromal cells, and the extracellular matrix. This detailed analysis allows the identification of specific cell types, molecular markers, and dynamic processes, loke oncogenesis, proliferation, and metastasis. Furthermore, scRNA-seq’s capacity to pinpoint rare resistant cells contributing to treatment failure makes it an invaluable techniques for developing targeted therapies. By enhancing the precision of cancer diagnostics and providing insights into novel therapeutic approaches, scRNA-seq is reshaping our strategies to this complex disease (Bridges and Miller-Jensen, 2022).

For example, scRNA-seq has been utilized to investigate T cell receptors in colorectal tumors, revealing subclass groupings, tissue organization, cancer variability, and gene expression related to drug responses. This capability solidifies scRNA-seq as an essential tool in developing new diagnostic and therapeutic strategies for cancer (Castellanos-Rueda et al., 2021). In another study, potential associations and transformations among T cell classes and subclasses within tissues were identified. Similarly, scRNA-seq was employed to explore genomic copy number variations, DNA methylation irregularities, and gene expression changes during colorectal tumor onset and progression, all at a single-cell level (Bian et al., 2018).

Furthermore, scRNA-seq is highly effective at detecting gene expression changes throughout cancer proliferation. By analyzing transcriptomes from individual cells in healthy tissues and adenomas at various progression stages, researchers have uncovered genomic variations, clonal structures, and metabolic instabilities involved in tumorigenesis. This approach provides valuable insights into cancer development (Li et al., 2021). For instance, Chen et al. highlighted that in the progression from familial adenomatous polyposis to adenocarcinoma, malignant cells retained epithelial characteristics while rapidly migrating, emphasizing the complexity of cancer evolution (Chen Y.-C. et al., 2019). In a study of treatment-resistant bladder cancer patients, single-cell transcriptomic sequencing was used to explore the cancer microenvironment, including immune cells, extracellular matrix, blood vessels, and fibroblasts (Lee et al., 2020). A similar approach was taken in renal cell carcinoma research, where cancerous tissues were compared with benign kidney tissues to better understand tumor progression and treatment responses (Zhang et al., 2021a).

Single-cell analysis plays a crucial role in identifying molecular control points and potential treatment targets within cancers, revealing insights often overlooked by traditional methods. By enabling the detailed examination of individual cancer cells, this approach allows researchers to pinpoint key genetic and epigenetic changes that drive tumor growth and resistance to therapies. One particularly powerful application is the genome-wide tracking of DNA mutations, which uncovers specific alterations that influence treatment efficacy. These mutations can identify vulnerabilities in cancer cells, aiding the development of targeted therapies that address the unique molecular profiles of individual tumors (Shalek and Benson, 2017). This approach is especially critical in cases where standard therapies fail, due to the complex and variable nature of individual tumors. Thus, single-cell transcriptomic sequencing holds great promise for advancing personalized treatment strategies (Mustachio and Roszik, 2022).

Beyond mutation tracking, single-cell investigation also offers insights into the regulatory networks and signaling pathways that govern cancer cell behavior. This includes detecting dysregulated genes, transcription factors, and other molecular mechanisms that could serve as potential intervention points. By mapping these control points, researchers can gain a better understanding of how cancers evolve and adapt, paving the way for more personalized and effective treatment mechanisms. Ultimately, single-cell sequencing technologies are not only advancing our understanding of cancer biology but are also transforming the precision and success of therapeutic intervention strategies (Wang R.-Q. et al., 2021; Chen et al., 2023).

### Implications in the area of immunology

Single-cell RNA sequencing (scRNA-seq) has revolutionized immunological research by offering detailed insights into the immune system. This powerful technology allows the investigations of particular immune cell types, the discovery of novel populations, and the tracking of their interactions within complex immune networks. By revealing the roles of specific immune cell subsets, scRNA-seq enhances our elucidations of immune responses in various conditions, such as infections, autoimmune diseases, and cancer. This knowledge is pivotal for developing targeted treatment approaches, like optimizing cancer immunotherapy and addressing chronic inflammation, paving the way for more precise and effective treatments (Kuksin et al., 2021). For instance, scRNA-seq has been employed to explore sub-populations of natural killer (NK) cells in both mice and humans. This research identified key characteristics that differentiate NK cells in the blood from those in the spleen and revealed two primary subclasses, NK1 and NK2, across different organs and species. These findings provide valuable insights into the biological roles of NK cells and improve the translation of animal research to human studies (Crinier et al., 2018).

Another notable application of scRNA-seq is in analyzing dendritic cells and monocytes in the human bloodstream. One study identified a new type of dendritic cell that shares features with plasmacytoid dendritic cells but exhibits a unique ability to activate T lymphocytes. This discovery highlights the potential of scRNA-seq to uncover previously unknown immune cell populations with critical functional roles (Villani et al., 2017). Furthermore, scRNA-seq is invaluable for studying immune responses during active infections. For example, research on IL-10-expressing CD4^+^ T cells demonstrated that variability in IL-10 production among helper T cells plays a vital role in enhancing humoral immunity across different diseases. This insight underscores the importance of single-cell approaches in understanding immune regulation and variability (Xin et al., 2018).

Beyond infectious diseases, scRNA-seq is instrumental in investigating immune cell variability induced by disease agents and other factors, such as aging. By enabling precise identification of the genetic and transcriptional profiles of individual immune cells, scRNA-seq provides unprecedented insights into the complexity of immune system functioning (Papalexi and Satija, 2018). Aging, for example, significantly impacts immune cell dynamics. A study utilizing scRNA-seq on CD4^+^ T cells from young and aged mice revealed that aging increases transcriptomic variability, leading to greater heterogeneity in gene expression within immune cells. This highlights how scRNA-seq can illuminate the molecular basis of age-related immune system changes (Martinez-Jimenez et al., 2017).

### Implications in the gastro-intestinal system and urinary tract system

Single-cell RNA sequencing (scRNA-seq) has become an indispensable techniques for examining the cellular complexity and functional dynamics of the gastrointestinal (GI) and urinary tract systems. In the GI system, scRNA-seq uncovers the varied functions of epithelial, immune, and stromal cells in critical processes including digestion, nutrient absorption, and immune defense. Additionally, it offers detailed insights into molecular changes associated with diseases like inflammatory bowel disease (IBD) and colorectal cancer. Likewise, in the urinary tract, scRNA-seq maps the intricate cellular composition of the kidney, bladder, and ureter, elucidating mechanisms involved in filtration, reabsorption, and urine formation. This technology also sheds light on cellular responses in pathological conditions like chronic kidney disease (CKD) and urinary tract infections (UTIs) (Haque et al., 2017; Li et al., 2021).

By capturing cellular heterogeneity and dynamic states, scRNA-seq significantly enhances our understanding of normal physiology and disease processes, driving advancements in precision medicine for both systems. For example, Haber et al. utilized scRNA-seq to identify novel subclasses of gut epithelial cells, providing insights into their roles in maintaining intestinal homeostasis and responding to pathogenic challenges (Haber et al., 2017). Additionally, Gao et al. applied high-resolution scRNA-seq to investigate gene regulatory processes in the digestive organs during human embryonic development and within adult large intestines. Their work has expanded knowledge on tissue-specific gene expression and developmental trajectories, offering valuable perspectives on organ function and maturation (Gao et al., 2018).

### Implications in the area of neurology

Single-cell RNA sequencing (scRNA-seq) has revolutionized neuroscience by revealing the cellular and molecular diversity within the nervous system. This cutting-edge technology enables precise mapping of neuronal and glial cell types, shedding light on their roles in brain development, function, and plasticity. In neurodegenerative diseases such as Alzheimer’s, Parkinson’s, and multiple sclerosis, scRNA-seq has uncovered disease-associated cell states, neuroinflammatory pathways, and potential biomarkers. Similarly, in psychiatric disorders like schizophrenia and autism, it provides critical insights into the mechanisms underlying neural circuit dysfunction. By offering a high-resolution view of the nervous system’s cellular landscape, scRNA-seq facilitates the discovery of novel therapeutic targets and supports the development of personalized approaches in neurology (Ofengeim et al., 2017; Cuevas-Diaz Duran et al., 2022).

The variability among individual neurons, often driven by specific copy number variations, poses challenges to understanding brain circuitry and neuronal connections. ScRNA-seq addresses these challenges by capturing different stages of neuronal differentiation and enabling the classification of neuron subtypes based on their molecular signatures. Advanced methods like single-cell methylation sequencing have further enhanced this understanding by redefining neuronal subclasses in both mouse and human frontal cortices through methylation pattern analysis (Xing et al., 2023).

In addition, innovative single-cell nuclear sequencing approaches have been applied to trace cell lineages within the adult brain. These methods have provided crucial insights into principal cell classes and their functional roles, deepening our comprehension of brain organization and cellular diversity. ScRNA-seq has also played a pivotal role in cerebellar development research, identifying key subsets of cerebellar cells and elucidating their roles in this complex process. Such findings lay the groundwork for future studies in neurobiology, offering new avenues for investigating neurological diseases and developmental disorders (Cardona-Alberich et al., 2021; Rosenberg et al., 2018).

### Implications in the area of reproductive and embryonic medicine

Single-cell RNA sequencing (scRNA-seq) plays a crucial role in studying small populations of cells, with significant applications in prenatal diagnostics and reproductive health. By analyzing egg cells and early embryonic stages, scRNA-seq helps select healthy embryos, potentially reducing the incidence of genetic disorders and preventing hereditary diseases in newborns (Hou et al., 2013; Li et al., 2017). This technology is especially valuable for investigating embryonic development, from the zygote stage to full maturity. It has transformed research in early mammalian development, shifting the focus from hypothesis-driven to discovery-driven approaches (Yan et al., 2013).

In model organisms like zebrafish and African cockroaches, scRNA-seq has provided key insights into cell growth and developmental biology (Briggs et al., 2018). Moreover, single-cell multi-sequence sequencing has been applied to map human embryos before implantation, uncovering the complex epigenetic processes involved in pre-implantation development (Alanis-Lobato et al., 2024). For example, Vento-Tormo et al. (Vento-Tormo et al., 2018) used scRNA-seq to analyze placental cells prior to gestation, creating detailed cell maps that identified sub-populations and key regulatory interactions crucial for successful placental development and modulating maternal immune responses.

In male reproductive biology, scRNA-seq has been used to trace gene expression during spermatogenesis, focusing on alternative splicing patterns and identifying key regulators involved in male germ cell development (Chen et al., 2018). The technology has also been applied to analyze both normal and diseased human testicular cells, uncovering hierarchical patterns of spermatogonial subclasses, spermatocyte subtypes, and sperm cell subclasses, along with specific markers for human germ cells. Additionally, scRNA-seq has revealed changes in expression profiles in testicular somatic cells from non-obstructive azoospermia (NOA) patients, providing new insights into the disease’s pathogenesis (Wang M. et al., 2018).

## Challenges in single-cell RNA sequencing technologies

Single-cell RNA sequencing (scRNA-seq) provides a detailed view of transcriptomic interactions within individual cells. However, several challenges remain with this technology. One major issue is limited capture efficiency; current scRNA-seq methods capture only a small fraction (approximately 10%) of each cell’s transcriptome, which leads to reduced sensitivity and difficulties in detecting low-abundance transcripts (Saliba et al., 2014; Deng et al., 2014). Additionally, the minimal input material required for scRNA-seq libraries introduces high technical noise, complicating data analysis and potentially obscuring true biological variations (Kolodziejczyk et al., 2015; Marinov et al., 2014). Efforts have been made to address technical noise by using spike-in controls to calibrate datasets. However, these techniques assume that spike-in transcripts behave stably, which may not always be the case since spike-in RNAs do not always mimic the behavior of cellular RNAs (Mendelevich et al., 2023).

Another challenge arises from the methods used to isolate and capture individual cells. Techniques such as micro-manipulation and laser dissection can be labor-intensive and require specialized equipment. More commonly, cells are separated from tissue samples to create a suspension for sequencing, but this process can impact cell viability and transcriptional status due to enzymatic treatments (Machado et al., 2021). To mitigate these issues, some studies have developed methods for performing RNA sequencing directly on individual nuclei, avoiding harsh enzymatic treatments (Grindberg et al., 2013). Despite significant advancements in scRNA-seq technology, limited capture efficiency and high technical noise continue to hinder accuracy and precision. As a result, ongoing efforts focus on improving experimental configurations and enhancing the reliability of single-cell RNA sequencing datasets (Ding et al., 2015).

Emerging *in situ* sequencing techniques provide an alternative approach to traditional RNA sequencing by enabling the capture and amplification of RNA within the intact tissue environment. These methods allow for RNA sequencing directly within cells, facilitating the generation of cDNA amplicons for rolling circle amplification and sequencing. While they offer the advantage of preserving the spatial context of the tissue, these techniques are currently limited in their ability to quantify only a few hundred genes per cell (Lee et al., 2015; Wang X. et al., 2018).

Furthermore, most scRNA-seq studies have primarily focused on polyadenylated mRNA, using poly-T priming techniques that capture only poly (A)+ transcripts. This approach limits the ability to explore non-polyadenylated RNA categories, such as regulatory non-coding RNAs or bacterial RNAs (Haile et al., 2021). To address this limitation, random hexamer priming has been proposed to capture both polyadenylated and non-polyadenylated transcripts. Additionally, computationally designed “not-so-random” primers may improve the capture of both poly(A)+ and poly(A)– species while minimizing ribosomal RNA contamination (Fitzpatrick et al., 2021).

## Discussion

Single-cell RNA sequencing (scRNA-seq) and spatial transcriptomics methods have revolutionized biomedical research by offering novel perspectives into cellular heterogeneity and structural organization of tissues. These technologies have significantly advanced our elucidation of complex biological systems, from developmental processes to disease pathogenesis. However, while these innovations hold great promise, numerous challenges and gaps remain, which need to be addressed to unlock their full capabilities (Longo et al., 2021; Ahmed et al., 2022).

Over the past few years, scRNA-seq has empowered scientists to discover the complex cellular diversity in tissues formerly regarded as uniform. This is particularly apparent in cancer studies, where scRNA-seq has uncovered rare tumor subpopulations responsible for drug resistance and metastasis. By mapping gene expression to tissue architecture, spatial transcriptomics provides an additional layer of resolution. Collectively, these advancements have laid the foundation for more precise disease models and personalized medicine. For instance, spatial transcriptomics has offered novel insights into the tumor microenvironment, allowing a better understanding of how cells communicate and influence tumor progression (Wang Q. et al., 2023; Du et al., 2023).

Despite these breakthroughs, challenges persist, with data interpretation and integration being a key hurdle. The complexity and volume of data generated by these technologies require sophisticated computational tools for analysis, but current methods are not always sufficient to capture the full scope of biological variability. Additionally, while scRNA-seq provides valuable insights into gene expression at the single-cell level, it lacks spatial context, which is critical for understanding cellular interactions within tissues. While spatial transcriptomics helps bridge this gap, the resolution and scalability of existing techniques remain constrained. Continued technological advancements are essential to enhance precision and throughput (Yan et al., 2024; Stuart et al., 2019).

In the future, several areas offer significant potential for research and technological progress. The creation of more robust and scalable platforms for single-cell and spatial transcriptomics will be key to broadening their application to larger cohorts and diverse disease models. Enhanced computational approaches that integrate single-cell data with spatial information will be crucial for unraveling complex biological systems *in vivo*. Furthermore, integrating scRNA-seq and spatial transcriptomics with other approaches, such as proteomics and imaging, could offer a more comprehensive view of cellular behavior in context. Standardized protocols and stringent quality control measures will also be crucial to ensure reproducibility and consistency across studies (Shen et al., 2022; Sang-Aram et al., 2024).

## Future perspectives and concluding remarks

Single-cell RNA sequencing (scRNA-seq) has emerged as a groundbreaking tool in the life sciences, offering valuable insights into biological processes and human diseases. Over the past decade, advancements in scRNA-seq technologies have made them more accessible and applicable across a wide range of life science fields. These innovations have paved the way for the creation of detailed single-cell atlases across different biological layers, enhancing our insight into gene and cell functions in both health and disease (Li et al., 2021). In the future, high-resolution maps from scRNA-seq will allow researchers to examine datasets more thoroughly, reducing the need for costly and labor-intensive specimen reprocessing. Innovations in microfluidics and combinatorial barcoding are expected to further enhance the scalability and cost-effectiveness of single-cell analyses (Chen T. N. et al., 2020).

The combination of scRNA-seq with other large-scale genetic technologies is poised to expand its capabilities. For example, integrating scRNA-seq with CRISPR-based genome-wide testing (such as Perturb-seq) enables the exploration of transcriptional factors through gene deletion. Tools like LinTIMaT, which merge single-cell transcript datasets with mutation information, also facilitate ancestry tracing (Zafar et al., 2020). As prime editing technologies advance, they will likely enhance our understanding of gene and cell functions, enabling extensive multi-omics analyses that assess gene regulatory mechanisms in both normal and pathological states (Choi et al., 2022).

Numerous successful and promising applications of integrating scRNA-seq with other techniques have emerged. For instance, combining scRNA-seq with spatial transcriptomics and multi-omics profiling has unveiled cancer cell heterogeneity, identified rare cell populations, and provided valuable insights into tumor progression and resistance. Combining scRNA-seq with spatial RNA mapping and single-cell imaging allows for the exploration of the spatial organization of brain cell types, aiding in the identification of novel disease-related subpopulations that could serve as new therapeutic targets. Furthermore, scRNA-seq combined with single-cell proteomics and spatial imaging provides a deeper understanding of immune cell interactions and dysregulation, which is crucial for the development of immunotherapies and vaccines. Lastly, integrating scRNA-seq with spatial transcriptomics in infection studies helps clarify tissue-specific responses to pathogens, enabling the identification of key factors that influence disease severity and host susceptibility, ultimately guiding antiviral drug and vaccine development (Zhang et al., 2021b; Bridges and Miller-Jensen, 2022).

Despite these advances, scRNA-seq still faces challenges in clinical applications. The high cost of sample preparation and sequencing remains a barrier to routine use. Additionally, the complexity of scRNA-seq data analysis, including data operation, visualization, and interpretation, highlights the need for the development of user-friendly, automated pipelines accessible to those without bioinformatics expertise. Addressing these challenges is crucial for expanding the clinical utility of single-cell transcriptomic sequencing (Brendel et al., 2022).

To conclude, single-cell RNA sequencing and spatial transcriptomics are reshaping biomedical research by enhancing our understanding of cellular function and tissue structure. Despite ongoing challenges related to data analysis, resolution, and scalability, continued advancements in these technologies hold the potential to open new paths for disease diagnosis, drug development, and personalized medicine. By overcoming these limitations and advancing these technologies, researchers can continue to make groundbreaking discoveries, paving the way for more effective and targeted therapies (Huang et al., 2024).

## Abbreviations

cDNA, Complementary Deoxyribonucleic Acid; CNV, Copy number variation; CRISPR, Clustered Regularly Interspaced Short Palindromic Repeats; DNA, Deoxyribonucleic Acid; GSVA, Gene set variation analysis; NK, Natural killer cell; NOA, Non-obstructive azoospermia; PCR, Polymerase chain reaction; RNA, Ribonucleic acid; scRNA-seq, Single-cell RNA sequencing; UMIs, Unique molecular identifiers.
